# Behavioral and Physiological Plasticity Provides Insights into Molecular Based Adaptation Mechanism to Strain Shift in *Spodoptera frugiperda*

**DOI:** 10.3390/ijms221910284

**Published:** 2021-09-24

**Authors:** Muhammad Hafeez, Xiaowei Li, Farman Ullah, Zhijun Zhang, Jinming Zhang, Jun Huang, Muhammad Musa Khan, Limin Chen, Xiaoyun Ren, Shuxing Zhou, G. Mandela Fernández-Grandon, Myron P. Zalucki, Yaobin Lu

**Affiliations:** 1 State Key Laboratory for Managing Biotic and Chemical Threats to the Quality and Safety of Agro-Products, Institute of Plant Protection and Microbiology, Zhejiang Academy of Agricultural Sciences, Hangzhou 310021, China; hafeez_203@yahoo.com (M.H.); lixiaowei1005@163.com (X.L.); zhijunzhanglw@hotmail.com (Z.Z.); zhanginsect@163.com (J.Z.); junhuang1981@aliyun.com (J.H.); clmit@zju.edu.cn (L.C.); renxiaoyunyouxiang@163.com (X.R.); 3090100232@zju.edu.cn (S.Z.); 2 State Key Laboratory of Rice Biology, Institute of Insect Sciences, Zhejiang University, Hangzhou 310058, China; 3 Department of Entomology, College of Plant Protection, China Agricultural University, Beijing 100193, China; farmanullah@cau.edu.cn; 4 Key Laboratory of Bio-Pesticide Innovation and Application, South China Agricultural University, Guangzhou 510642, China; drmusakhan@outlook.com; 5 State Key Laboratory of Ecological Pest Control for Fujian and Taiwan Crops, Key Lab of Biopesticide and Chemical Biology, Ministry of Education & Fujian Province Key Laboratory of Insect Ecology, College of Plant Protection, Fujian Agriculture and Forest University, Fuzhou 350002, China; 6 Natural Resources Institute, University of Greenwich, Chatham Maritime, Kent ME4 4TB, UK; m.fernandez-grandon@greenwich.ac.uk; 7 School of Biological Sciences, University of Queensland, Brisbane, QLD 4072, Australia; m.zalucki@uq.edu.ua

**Keywords:** midgut, antennal response, host plants adaptation, molecular mechanism, *Spodoptera frugiperda*, behavioral response

## Abstract

How herbivorous insects adapt to host plants is a key question in ecological and evolutionary biology. The fall armyworm, (FAW) *Spodoptera frugiperda* (J.E. Smith), although polyphagous and a major pest on various crops, has been reported to have a rice and corn (maize) feeding strain in its native range in the Americas. The species is highly invasive and has recently established in China. We compared behavioral changes in larvae and adults of a corn population (Corn) when selected on rice (Rice) and the molecular basis of these adaptational changes in midgut and antennae based on a comparative transcriptome analysis. Larvae of *S. frugiperda* reared on rice plants continuously for 20 generations exhibited strong feeding preference for with higher larval performance and pupal weight on rice than on maize plants. Similarly, females from the rice selected population laid significantly more eggs on rice as compared to females from maize population. The most highly expressed DEGs were shown in the midgut of Rice vs. Corn. A total of 6430 DEGs were identified between the populations mostly in genes related to digestion and detoxification. These results suggest that potential adaptations for feeding on rice crops, may contribute to the current rapid spread of fall armyworm on rice crops in China and potentially elsewhere. Consistently, highly expressed DEGs were also shown in antennae; a total of 5125 differentially expressed genes (DEGs) s were identified related to the expansions of major chemosensory genes family in Rice compared to the Corn feeding population. These results not only provide valuable insight into the molecular mechanisms in host plants adaptation of *S. frugiperda* but may provide new gene targets for the management of this pest.

## 1. Introduction

How plant-feeding insects adapt to new hosts is a key process in their diversification. For phytophagous insects, the process of selecting a host plant is complex and is the result of millions of years of coevolution between plants and animals [1]. Adaptation to host plants occurs through new mutations or through existing genetic variation in the population [2,3]. Occasional expansion of the host range, as well as the direct role of plant related adaptation leading to effective reproductive isolation between host-specialized populations, are increasingly recognized as factors in the diversification of herbivores [4,5,6,7]. However, little is known about the evolutionary processes leading to such host range expansions, since the genetic basis of host plant adaptation mechanisms is not well understood.

Herbivorous insects are engaged in a very intimate and challenging relationships with their host plants. Plants are the key components of the ecological niche for herbivorous insects being a food resource, mating site, egg-laying site and habitat essential for the insects survival and reproduction [8,9,10,11]. Therefore, the use of plant hosts involves key morphological, behavioral and physiological adaptations that are essential for development and survival [9,10,12,13,14]. Molecular based studies of the mechanisms involved in adaptation to host plants have emphasized the importance of key genes relating to sensing, digestion and detoxification [15]. In lepidopterans, different groups of genes related to plasticity and host adaptation have been described: principally an extended family of genes encoding olfactory receptors (ORs) and gustatory receptors (GRs) potentially involved in host sensing [16,17,18,19]. Even though increasing numbers of ORs and GRs from many lepidopteran species have been found [16] the exact genetic basis behind the preferences for specific hosts have not yet been clarified. Early studies focused on insect olfactory plasticity and modulation mechanisms in the central nervous system (CNS) [20], while recent studies have shown modulation at the olfactory receptors (ORs) within the olfactory neurons (OSNs) on antennae as a function of odor exposure [21,22,23].

Adaptation of an insect to a particular host-plant largely depends on their digestive physiology being able to handle chemically diverse plants as a food source [24,25,26]. In most cases, this generally involves the induction of a cocktail gut digestive enzymes that allow the exploitation of the toxic phytochemicals encountered during feeding [24,27]. The expression of gut digestive plasticity to cope with plant chemical toxicants as an adaptive mechanism to noxious chemical containing host plants has been documented in a number of herbivorous insects including *Busseola fusca* [28], *Helicoverpa armigera* [29], *Spodoptera frugiperda* [30], *Heliothis virescens* [31] and *Manduca sexta* [32]. In herbivorous insects, digestive proteases with diverse structures and functions play an important role in host plant adaptation. The highly expressed activity of cytochrome P450s, glucosinolate sulfatases and carboxylesterases are potentially involved in detoxification of various plant defensive compounds or xenobiotics and a mechanism in host plant adaptation in herbivorous insect [13,17,33]. Recent work has provided evidence that polyphagous insects can respond to secondary metabolites produced by host plants by induced changes in gene expression related to detoxification and digestion enzymes that provide greater fitness on a specific host [34,35,36,37,38]. Currently RNA-Seq, one of the most widely used next-generation sequencing technology, is providing a wider dynamic range and better estimates of absolute gene expression levels. This technology has been used to elucidate the types and functions of large gene families related to sensing, digestion and detoxification involved in host plant utilization in various insect species [24,34,39,40].

The fall armyworm (FAW), *Spodoptera frugiperda*, is highly invasive [41] and one of the major agronomically important pest in the Americas, causing severe damage to economically important crops. It is considered a polyphagous species, being variously reported on 100–300 odd host plants from at least 27 families [42,43]. The species has invaded Africa, Asia and Australia and spread rapidly within all these continents, triggering serious agricultural production and economic losses [43,44,45,46,47,48]. Even though *S. frugiperda* is considered a single species two-host plant-related strains have been described; a Corn strain (CS) that prefers to feed on maize (corn) and sorghum, and a Rice strain (RS) that feeds on rice and in particular Bermuda grass (Ribeiro et al., 2020). Even though there is no significant morphological difference between the strains they can be distinguished genetically by strain-specific molecular markers [49,50,51].

Here we investigate the genomic plasticity of the corn strain of FAW when confronted by the alternate plant (rice) after 20 generations of selection. We conducted phenotypic bioassays over 20 generations in the context of feeding choice (FD) and oviposition choice (OV) to the originally preferred corn and alternate rice plants, during which we measured fitness associated traits to estimate the comparative preference-performance of the ‘strains’ on both plants. To determine how the corn strain adapts to rice during larval feeding and adult oviposition the transcriptome profiles of midguts of 5th instars larvae and antennae of adults (Male and Female) were compared, at the gene expression level. This was carried out for the corn strain population reared in corn (Corn-population) and corn population on rice (Rice-population) for 20 generations and corn population on rice plants for one generation (C-R) (after 20 generation the corn population was fed on rice for one generation C-R). We searched for molecular mechanism and genes consistently differently transcribed between the two populations to determine which might be linked to adaptive differences.

## 2. Materials and Methods

### 2.1. Insect Collection and Rearing

Populations of *S. frugiperda* larvae were originally collected from two different corn fields in Ping Hu County (Latitude: 30.705° N, Longitude: 121.118° E), Zhejiang Province during August 2019 and initially established on corn plants in a climatically controlled chamber at 25 ± 2 °C with a 14:10 h light: dark photoperiod in the Zhejiang Academy of Agricultural Sciences, Hangzhou, China. Following pupation, newly emerged moths were paired and placed together in mating cages and provided 10% honey solution as a food source. The population was reared through one generation on 15–20 days old corn plants before being used in host selection experiments. Late larval instars and adults were inspected to confirm species based on diagnostic taxonomic characters and using the strain-specific *Tpi* molecular marker [52].

### 2.2. Rearing and Host Plant Selection

The seeds of two commercially available host plants; rice (Liangyou-887), *Oryza sativa* and sweetcorn (Zhetaitian928), *Zea mays* were purchased (Gan Su Dunhuang Seed Co., ltd 28 Suzhou Rd Jiuquan Gansu province China) and planted in vermiculite mixed with peat moss (4:1) in plastic trays: 38 × 38 × 8 cm for corn and 40 × 30 × 8 cm for rice. The rice plants were grown in a controlled plant growth chamber at 28 ± 2 °C with a 14:10 h: light and dark photoperiod and corn plants were grown in a greenhouse with the same growth condition at Zhejiang Academy of Agricultural Sciences. Plants 15–20 days old were used for all generations and experiments.

Two populations of *S. frugiperda* were established, designated as Corn-pop and Rice-pop, from the original population by rearing each continuously on their respective plants. Both populations were reared according to the method described by [30] for 20 generations.

### 2.3. Larval Feeding Choice in Y-Tube Olfactometers

**Figure 1:**
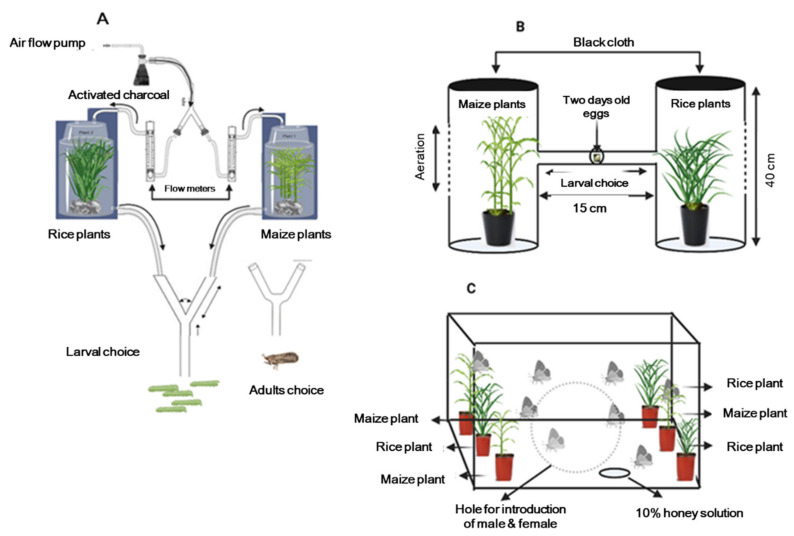
Graphical representation of choice apparatus used for larval feeding choice and adults oviposition preference behavior. (**A**) Y tube olfactometer set up for neonates and adults, (**B**) Choice chamber for neonates and (**C**) Cage set up for oviposition assays.

The olfactory orientation responses of first instar FAW from generation 1–20 to rice and corn was performed in a Y-tube olfactometer at 27 ± 2 °C [53]. The experimental choice arena consisted of a glass y-tube (7.5 cm for each arm and base, 15 cm total length, 10 mm i.d., 12.7 mm o.d.; Chemglass, Inc., Vineland, NJ, USA) with two transparent plastic tubes attached separately at the distal end of each arm and the other ends connected to the top of a glass jar containing either corn or rice. Plants were 15–20 days taken with roots and leaves washed with 70% ethanol followed by thorough rinsing with Milli-Q-filtered water and the roots wrapped with aluminum foil covering 2 cm up the stem (Figure 1A). The air flow from the olfactometer was introduced at the bottom of each jar through transparent plastic tube. Total airflow was maintained and monitored at 300 mL/min through each arm (0.12 km/h) and 300 mL/min (0.18 km/h) out of the base of the y-tube by a flow meter (Aalborg Instruments, Orangeburg, NY, USA)-regulated Tygon vacuum line. All parts of the Y-tube apparatus between the plants source and vacuum line were cleaned or replaced with new one between bioassay trials to remove contaminants.

Since FAW is primarily nocturnal in its peak-feeding activity, all bioassays were conducted in a dark room. Early first instars that had emerged within the previous 12 h were used in the Y-tube bioassays. A total of 20 first instar larvae were introduced into the Teflon tube union between the Y-tube base and vacuum line as described above. The larvae were then given 20 min to make a choice between the plant odors emanating from each arm. Larvae that remained motionless for 10 min were presumed damaged by handling and were replaced. Similarly, larvae that moved but were not be able to make a choice within 20 min were recorded as neutral or non-responders and were excluded from the experiment. Separate experiments were performed for corn and rice population in every generation. For each experiments a total of 100 first instar larvae were used from each population with five replicates (each replicate consisted of 20 neonates). Each experiment was performed in triplicates.

### 2.4. Larval Feeding Choice Bioassays on Whole Plants in Cages

Orientation and feeding assays (Figure 1B) were performed with first instar *S. frugiperda* larvae originating from adults previously reared on each of the two host plants at 27 ± 2 °C. We performed two choice feeding experiments in plastic boxes (30 cm high × 15 cm square base) connected with a 15 cm tube. A corn or rice plant was centered inside each plastic box to make a two choice feeding arena and the upper part of each box covered with black cloth for aeration and to avoid larvae escaping. Approximately 50 two-day old eggs taken from a number of egg batches laid by females from each population, Corn-pop or Rice-pop, were placed at the center of the tube that connected the two boxes via a hole (Figure 1B), the hole was closed with paraffin and the tube covered with black cloth to avoid direct light which could affect the larval movement. After four days, larvae were counted from each box either containing the corn or rice plants. This experiment was performed in triplicate from each population (Corn-pop and Rice-pop) at every generation.

### 2.5. Female Adult Host Preference Experiment in Y-Tube Olfactometer

The olfactory choice response of female FAW moths to rice and corn was performed in a Y-tube olfactometer as described above with some modifications at 27 ± 2 °C. The experimental choice arena consisted of a glass Y-tube (7.5 cm for each arm and base, 15 cm total length, 10 mm i.d., 12.7 mm o.d.; Chemglass, Inc., Vineland, NJ, USA. Host preference of *S. frugiperda* female adult reared on either corn or rice plants were compared. All bioassays were conducted in a dark room. One day old female moths were used in the bioassays. In this case, 10 moths were introduced into the Teflon tube union between the Y-tube base and vacuum line as described above. The moth was given 40 min to make a choice between the plant odors emanating from each arm. Moths that remained motionless and were not able to make a choice within 40 min were recorded as neutral or non-responders and were excluded from the experiment. Separate experiments were performed for corn and rice population in every generation. A total of 30 female moths were used for each population in three replicates (each replicate consisted of 10 female moths). 

### 2.6. Female Oviposition Choice Experiments of Fall Armyworm in Cages

Oviposition choice of female moths to corn and rice were observed in oviposition cages (60 cm cube) at 27 ± 2 °C in a control chamber. Briefly, three pots containing 15–20 d seedling corn and three containing 15–20 days rice plants (10 cm apart from each other) were placed inside the cage (Figure 1C). Five pairs of one day old Corn-pop moths (5 ♀ + 5 ♂) were released for mating and oviposition in each cage. The same set-up was used for the Rice-pop. The oviposition choice of females was observed every day by counting and removing all eggs laid by female for 10 d on corn and rice plants. Each experiment was replicated three times for each population in each generation.

### 2.7. Larval Performance Experiments

Larval performance of each population was evaluated by rearing on corn or rice plants and measuring the larval developmental time (at 20 generation) and pupal weight (in every generation) at 27 ± 2 °C in a control chamber. For neonates to third-instar larvae, 15-days old rice and corn seedling were provided, whereas 20-days old rice or corn plants were provided for fourth- and sixth-instar larvae. Approximately 50 newly hatched first instar larvae (taken from a mixture of eggs masses) were used from each population and released in separate rearing cages (15-cm high, 10-cm diameter, cylindrical polyethylene terephthalate cages with nylon mesh cloth). Populations were observed daily and all data recorded until pupation.

### 2.8. Sample’s Preparation, RNA Extraction, Library Preparation and Sequencing

A total of 180 adults (30 females and 30 males) were selected from three populations, Corn-pop, Rice-pop after 20 generations and a Corn-Rice population after one generation (larvae reared on corn for 20 generations and transferred onto rice plants for one generation were designated as corn population on rice). The antennae of moths from each treatment were removed, put in Eppendorfs, frozen in liquid nitrogen immediately and stored at −80 °C until RNA extraction. Similarly, a total of 90 fifth instar larvae (30 larvae from each treatment) were selected from each treatment, midguts removed and contents washed with 0.9% NaCl physiological solution to remove residual food, then immediately immersed in liquid nitrogen and stored at −80 °C until RNA extraction. There were three independent biological replicates for each treatment (each replicate was consisted of 10 midguts and 10 antennae).

### 2.9. RNA Quantification and Qualification RNA Integrity

RNA quantification and integrity were assessed using the RNA Nano 6000 Assay Kit of the Bioanalyzer 2100 system (Agilent Technologies, Santa Clara, CA, USA).

### 2.10. Library Preparation for Transcriptome Sequencing

One microgram RNA/ sample was used as input material for the RNA sample preparations. In brief, mRNA was purified from total RNA using poly-T oligo-attached magnetic beads. Fragmentation was carried out using divalent cations under elevated temperature in First Strand Synthesis Reaction Buffer (5×). First strand cDNA was synthesized using random hexamer primer and M-MuLV Reverse Transcriptase (RNase H-). Second strand cDNA synthesis was subsequently performed using DNA polymerase I and RNase H. Remaining overhangs were converted into blunt ends via exonuclease/polymerase activities. After adenylation of 3′ ends of DNA fragments, Adaptor with hairpin loop structure were ligated to prepare for hybridization. In order to select cDNA fragments of preferentially 370~420 bp in length, the library fragments were purified with AMPure XP system (Beckman Coulter, Beverly, CA, USA). Then PCR was performed with Phusion High-Fidelity DNA polymerase, Universal PCR primers and Index (X) Primer. At last, PCR products were purified (AMPure XP system) and library quality was assessed on the Agilent Bioanalyzer 2100 system.

### 2.11. Reads Mapping to the Reference Genome

Reference genome and gene model annotation files of *S. frugiperda* were directly downloaded from genome website (http://bipaa.genouest.org/is/lepidodb/spodoptera_frugiperda). An index of the reference genome was built using Hisat2 v2.0.5 and paired-end clean reads were aligned to the reference genome using Hisat2 v2.0.5. We selected Hisat2 as the mapping tool as Hisat2 can generate a database of splice junctions based on the gene model annotation file and thus a better mapping result than other non-splice mapping tools.

### 2.12. Novel Transcripts Prediction

The mapped reads of each sample were assembled by String Tie (v1.3.3b) (Mihaela Pertea et al., 2015) in a reference-based approach. String Tie uses a novel network flow algorithm as well as an optional de novo assembly step to assemble and quantity full-length transcripts representing multiple splice variants for each gene locus.

### 2.13. Quantification of Gene Expression Level

Feature Counts v1.5.0-p3 was used to count the number of reads mapped to each gene. The Fragments Per Kilobase of transcript sequence per Millions FPKM of each gene was calculated based on the length of the gene and reads mapped to this gene counted. An expected number of FPKM base pairs sequenced, considers the effect of sequencing depth and gene length for the reads count at the same time, and is currently the most commonly used method for estimating gene expression levels.

### 2.14. Differential Expression Analysis

Differential expression analysis of two conditions (two biological replicates per condition) was performed using the DESeq2 R package (v1.20.0). DESeq2 provides statistical routines for determining differential expression in digital gene expression data using a model based on the negative binomial distribution. The resulting *p*-values were adjusted using the Benjamini and Hochberg’s approach for controlling the false discovery rate. Genes with an adjusted *p*-value < 0.05 found by DESeq2 were assigned as differentially expressed.

### 2.15. GO and KEGG Enrichment Analysis of Differentially Expressed Genes

Gene Ontology (GO) enrichment analysis of differentially expressed genes was implemented by the cluster Profiler R package, in which gene length bias was corrected. GO terms with corrected value less than 0.05 were considered significantly enriched by differentially expressed genes. KEGG is a database resource for understanding high-level functions and utilities of gene expression in biological systems, such as the cell, the organism and the ecosystem, from molecular-level information, especially large-scale molecular datasets generated by genome sequencing and other high-through put experimental technologies (http://www.genome.jp/kegg/). We used cluster Profiler R package to test the statistical enrichment of differentially expressed genes in KEGG pathways.

### 2.16. Weighted Correlation Network Analysis

Weighted correlation network analysis (WGCNA) was used to describe the gene association modes among different samples. It can be used to identify gene sets that show highly synergistic changes and identify candidate biomarkers or therapeutic targets based on the coherence of gene sets and the correlation between gene sets and phenotypes. The R package WGCNA is a set of functions used to calculate various weighted association analysis, which can be used for network construction, gene screening, gene cluster identification, topological feature calculation, data simulation and visualization. WGCNA is suitable for multisampling data. Generally, more than 15 samples are required.

### 2.17. Validation of Transcriptome Data Using RTq-PCR and Data Analysis

The relative expression levels of selected genes obtained from transcriptome analysis were validated using RT-qPCR and CFX connect TM Real-Time System (Bio-Rad, Hercules, CA, USA) was used for expression pattern. Total RNA of the midguts and antennae was used from our transcriptome analysis and cDNA was synthesized using *TransScript*^®^ One-step gDNA Removal and cDNA Synthesis SuperMix (transgenebiotech.com, No. 1 North Yongtaizhuang Road, Haidian District, Beijing, China 100192). The primers of selected genes were designed using the predicted CDSs as reference sequences. All the primers used in this study are listed in Table S3. SsoFast Evergreen^®^ Supermix Kit (Bio-Rad) was used to prepare the RT-qPCR reaction, following the manufacturer’s instruction. The qPCR cycling parameters were as follows: 95 °C for 3 min, followed by 40 cycles of 95 °C for 15 s and 57–60 °C for 30 s, melt curves stages at 95 °C for 15 s, 60 °C for 30 s. Reproducibility was checked by including a negative controls without template in each experiment, and three technical replicates and three biological replicates were used for each sample to performed the qPCR reaction. The relative expression of mRNA was calculated using the 2–ΔΔCt method [54]. Two reference genes, GAPDH and S30 were used for data normalization. All data was analyzed using the SPSS Statistics v24.0 software (IBMSPSS Statistics Inc., Chicago, IL, USA) and all obtained results were reported as mean ± SE.

### 2.18. Statistical Analysis

All data of FAW related to larval performance, Larval feeding choice, pupal weight, Female adult host preference, females’ oviposition and RTq-PCR was analyzed using an ANOVA with differences identified using the Tukey multiple comparison post hoc test at *p* ≤ 0.05. The SPSS 20.0 Software Package (SPSS Inc., Chicago, IL, USA).

## 3. Results

### 3.1. Feeding Choice of Fall Armyworm on Preferred and Alternate Host Plants in Y-Tube Olfactometer

**Figure 2:**
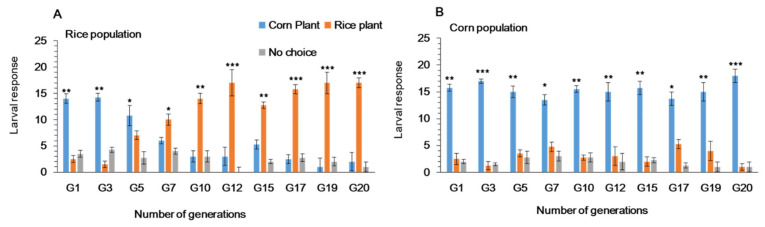
Feeding choice of first instars larvae of *Spodoptera frugiperda* in Y-tube, when reared on (**A**) rice and (**B**) corn plants for 20 generations. Data are means ± SE. Asterisks *, ** and *** show significant differences in different host plants (*p* < 0.05) using Tukey multiple comparison post hoc test at *p* ≤ 0.05.

The Y-tube olfactometer indicated difference in responses to plant odors between the two populations (Figure 2). Initially, the first instar larvae of fall armyworm reared on rice plants showed a significant response to corn odors but changed to rice around G6-G7 (Figure 2A). First instar larvae of fall armyworm reared on primary host plant (Corn-pop) for more than 20 generations, consistently oriented to corn plant odors as compared to rice (Figure 2B).

### 3.2. Feeding Choice of Fall Armyworm on Preferred and Alternate Host Plants in Plastic Cages

**Figure 3:**
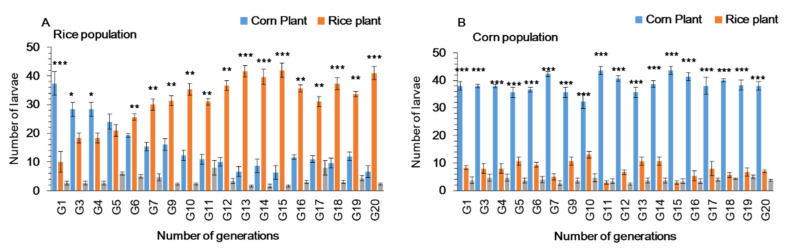
Feeding choice of first instars larvae of *Spodoptera frugiperda in* plastic cages to whole plants, when reared on (**A**) rice and (**B**) corn plants for 20 generations. Data are means ± SE. Asterisks *, ** and *** show significant differences between host plants (*p* < 0.05) based on post hoc Tukey’s HSD test.

First instar larvae of fall armyworm reared on rice plants (Rice-pop) continuously for 20 generations showed a feeding preference toward the alternate host plant (rice) as compared to corn (Figure 3A) with the change occurring after 6 generations. As expected, first instar larvae of fall armyworm maintained on the primary host plant (Corn-pop) showed a feeding preference toward corn (Figure 3B). In the Rice-pop the highest feeding preference for rice was observed at generation 13, 14, 15 and 20 (Figure 3B).

### 3.3. Larval Performance of Fall Armyworm on Preferred and Alternate Host Plants

**Figure 4:**
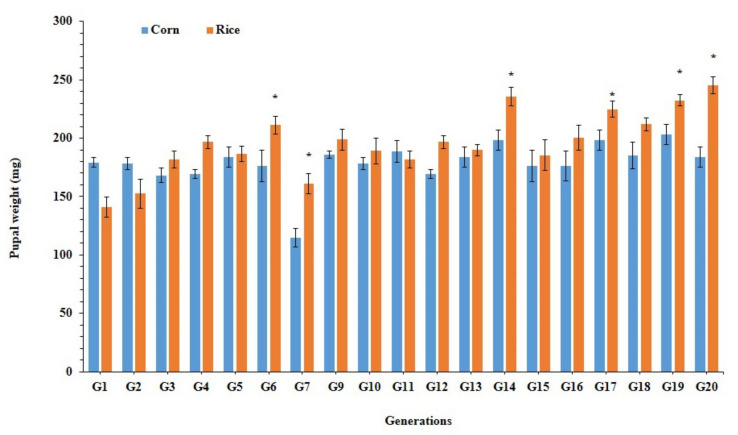
Average pupal weight (±SE) of *Spodoptera frugiperda*, when reared on corn and rice plants for 20 generations. Asterisks * show significant differences between hosts plants (*p* < 0.05) based on post hoc Tukey’s HSD test of the Corn-Pop reared on corn vs. the Rice-Pop reared on rice.

Larval duration was significantly shorter on rice population at generation 20 (Rice-pop-G-20) as compared to rice population at generation-1 (Rice-pop-G-1) and corn population (Corn-pop-G-20) (Table 1). After 20 generations of feeding on preferred (corn) and alternate (rice) host plants, the developmental time ranged from 14.2 to 19.8 days in the rice population, which was significantly shorter than in corn population (16.7 to 20.5 days) and rice population at generation-1 (18.3 to 24.3 days). The survival rate was significantly higher on rice population (Rice-pop-G-20) as compared to rice population at generation-1 (Rice-pop-G-1) and corn population (Corn-pop-G-20) (Table 1). Pupal weight was significantly different for rice population and corn population from 1–20 generations; corn population pupae were significantly heavier than rice population pupae during the initial generations. Rice population pupae were significantly heavier than the pupae of corn population after 4–20 generations (Figure 4).

### 3.4. Female Oviposition Choice of Fall Armyworm in Cages

**Figure 5:**
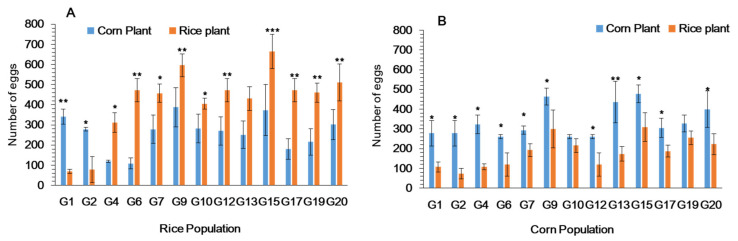
Total number of eggs laid by *Spodoptera frugiperda* adults on rice and corn plant when larvae reared on (**A**) rice and (**B**) corn plants for 20 generations. Data are means ± SE. Asterisks *, ** and *** show significant differences between host plants (*p* < 0.05) based on post hoc Tukey’s HSD test.

Host choice for oviposition was not significantly different in fall armyworm females when reared on preferred corn and alternate rice from generations 1–3; females laid more eggs on corn. Subsequently females from rice population laid more eggs on rice plants (Figure 5A). Female from the corn population consistently laid more eggs on corn except in G10 (Figure 5B). The highest number of eggs were observed on the alternate host (rice) at generation 9 and 15, respectively (Figure 5).

### 3.5. Host Plants Preference of Female for Oviposition Choice in Y-Tube Olfactometer

**Figure 6:**
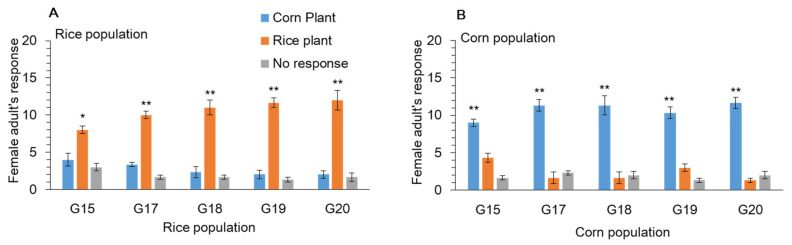
Response of *Spodoptera frugiperda* female to rice and corn plants in Y-tube, when reared on (**A**) rice and (**B**) corn plants for 20 generations. Data are means ± SE. Asterisks * and ** show significant differences in different host plants (*p* < 0.05) based on post hoc Tukey’s HSD test.

Olfactometer tests confirmed cage choice experiments. Initially orientation was to corn in both populations (data not shown). However, adult females from the rice population showed significant orientation to rice as compared to corn population females when larvae continued to be reared on rice (Figure 6A) compared to corn (Figure 6B) after 15–20 generation of selection.

### 3.6. RNA Sequencing and de novo Assembly

The total number of clean reads varied among different replicates. A total of 830,461,708 clean reads from male and female antennae and 403,714,968 clean reads from larval midguts were obtained. The quality of sequencing among different replicates did not differ greatly, the Q20 and Q30 of all samples was more than 90–95% with GC ratio more than 45% (Tables S1 and S2).

### 3.7. Differential Expressed Genes (DEGs) When Reared on Rice and Corn Plants

**Figure 7:**
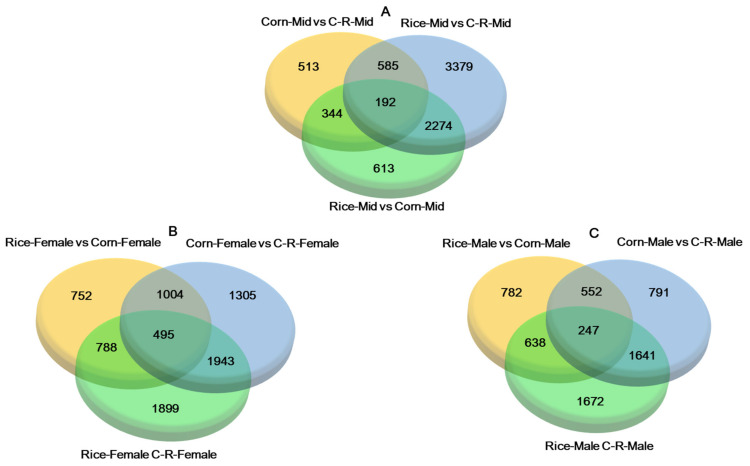
Venn diagrams depicting overlap among differentially expressed genes (log2 (FC) ≥ 1, FDR < 0.05) from pairwise comparisons among the three populations of *Spodoptera frugiperda*. The number of co-expressed unigenes in the Corn-midgut vs. C-R-Midgut, Rice-midgut vs. C-R-Midgut and Rice-midgut vs. Corn-Midgut (**A**), Rice-female vs. Corn-female, Corn-female vs. C-R-female (After 20 generation the corn population was fed on rice for one generation (C-R-female)) and Rice-female vs. C-R-female (**B**) and Rice-male vs. Corn-male, Corn-male vs. C-R-male and Rice-male vs. C-R-male antennae (**C**).

To better investigate the biological mechanism of adaptation to different host plants in *S. frugiperda* we identified similarities and differences in DEGs between the two populations with different treatments. The transcriptome analysis compared the number of common and unique genes (unigenes) between larval midguts (Mid), when *S. frugiperda* larvae fed on preferred (Corn), the alternate host plants (Rice) for 20 generations and Corn to Rice (C-R) for one generation (Figure 7A). Of 6430 highly expressed DEGs in Rice-Mid vs. C-R-Mid 3140 were up-regulated and 3290 were down-regulated (Figure S1A). Most of the differentially expressed genes (by at least two-fold) related to digestion (trypsin); of 102 DEGs 45 were up-regulated and 57 were down-regulated; and detoxification (P450s); of 56 DEGs 34 up-were regulated and 22 were down-regulated (Table 2). The co-regulation of DEGs from the antennae of different treatments (Figure 7B,C) shows that of 5125 highly expressed DEGs in Rice-Females vs. C-R-Females 2554 were up-regulated and 2571 were down-regulated (see also Figure S1B). Similar trend was found in Rice-Males vs. C-R-Males. Most of the differentially regulated genes related to PBP/GOBP family with 21 up-regulated and 4 DEGs down-regulated (Table 3).

### 3.8. Gene Ontology (GO) Enrichment Analysis of Midgut

**Figure 8:**
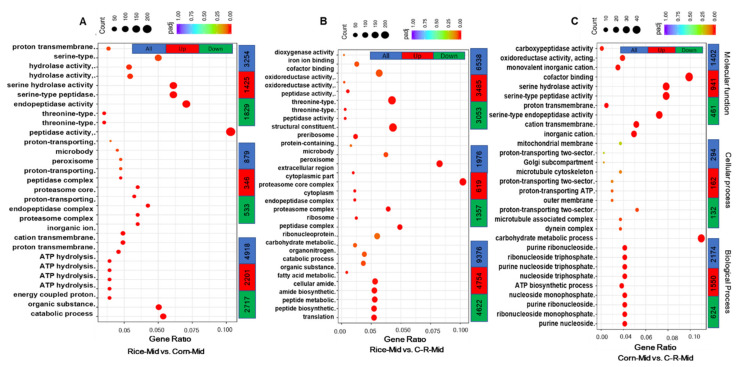
Gene Ontology (GO) classification of transcripts. The number of significantly up- and down-regulated unigenes are assigned into three main ontologies: biological process, cellular component and molecular function in the Rice-Mid vs. Corn-Mid (**A**), Rice-Mid vs. C-R-Mid (**B**) and Corn-Mid vs. C-R-Mid (**C**) (After 20 generation of selection the corn population was fed on rice for one generation (C-R-midgut)) of *Spodoptera frugiperda* when reared on corn and rice plants.

Our results indicate a total of 9051 differentially expressed unigenes in gene ontology (GO) annotation in Rice-Mid vs. Corn-Mid. Among three main ontologies those unigenes related to biological processes were highest (4918 or 54.3%), followed by molecular function (3254, 35.6%) and cellular component (879, 0.97%) (Figure 8A). Similarly, the number of contigs differently expressed in Rice-midgut vs. C-R-midgut was highest in this pairwise comparison contrasted with all others, a total of 17,890 differentially regulated unigenes, with 9376 (52.4%) in biological process, 6538 (36.5%) in molecular function and 1976 (0.11%) in cellular processes (Figure 8B). Similar trend was observed in Corn-mid vs. C-R-Mid (Figure 8C). Majority of the DEGs were related to oxidoreductase activity, hydrolase activity and cofactor binding.

### 3.9. Gene Ontology (GO) Enrichment Analysis of Male and Female Antennae

**Figure 9:**
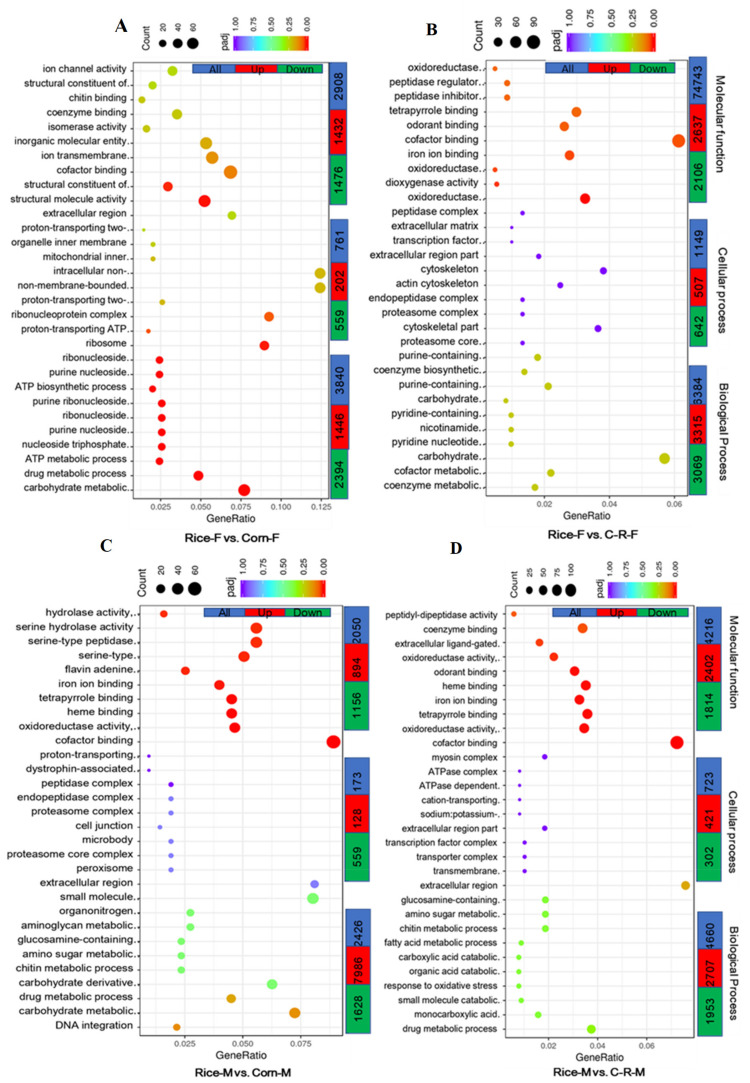
Gene Ontology (GO) classification of transcripts of Female (F) and Male (M) antennae. The number of significantly up- and down-regulated unigenes are assigned into three main ontologies: biological process, cellular component and molecular function in the Rice-F vs. Corn-F (**A**), Rice-F vs. C-R-F (**B**), Rice-M vs. Corn-M (**C**) and Rice-M vs. C-R-M antennae (**D**) (After 20 generation of selection the corn population was fed on rice for one generation (C-R-male)) of *Spodoptera frugiperda* when reared on corn and rice plants for 20 generations.

A total of 7509 unigenes were differentially expressed in Rice-Female vs. Corn-Female group and classified into the three main ontologies: biological process was the largest class with a total of 3840 (51.1%) unigenes; 1446 (19.3%) up and 2394 (31.9%) downregulated; followed by molecular function with 2908 (38.7%) unigenes and cellular component class with 761 (10.1%) up or down regulated (Figure 9A). The majority of DEGs in the GO analysis that were enriched related to biological process such as carbohydrate metabolism, drug metabolism and nucleoside triphosphate metabolism. Similarly, the two subcategories of DEGs most enriched in molecular function were related to cofactor binding and ion transmembrane transporter activity (Figure 9A). Similarly, of the 12,276 unigenes up or down regulated in Rice-Female vs. C-R-Female, the largest class was related to molecular function (52%) such as cofactor binding and odorant binding with 3315 (27%) up and 3069 (25%) down-regulated, followed by biological process with a total of 4743 (38.6%) significant DEGs related to carbohydrate metabolic process 1149 (9.4%) unigenes in the cellular component class (Figure 9B). In Rice-Male vs. Corn-Male comparisons 4777 unigenes were up or down regulated and again biological process was the most influenced class with a total of 2426 (50.8%) unigenes of which 798 (16.7%) were up-regulated and 1628 (34.1%) down-regulated and were related to carbohydrate metabolic (GO:0005975) and carbohydrate derivative metabolic process (GO:1901135). Of 2050 (42.91%) unigenese related to molecular function 894 (18.7%) were up- and 1156 (24.3%) down-regulated with cofactor binding containing the most significant DEGs. Cellular component was the least modified class with 31 (6.3%) (Figure 9C). A similar trend was observed in Rice-Male vs. C-R-Male (Figure 9D).

### 3.10. KEGG Pathway Enrichment Analysis of Midguts

**Figure 10:**
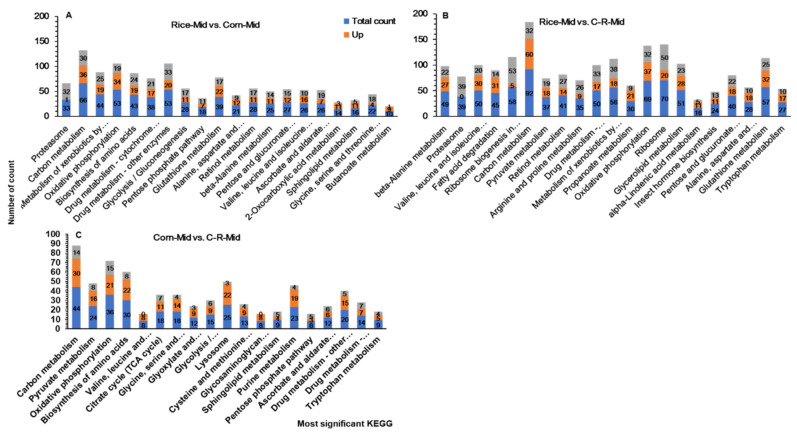
Enriched KEGG pathways for up and down regulated DEGs and the ratio of the DEG number to the total gene number in a certain pathway in the Rice-Mid vs. Corn-Mid (**A**), Rice-Mid vs. C-R-Mid (**B**), corn-Mid vs. C-R-Mid (**C**) (After 20 generation of selection the corn population was fed on rice for one generation (C-R-midgut) of *Spodoptera frugiperda* when reared on corn and rice plants for 20 generations.

KEGG analysis revealed significant transcriptional responses and enzymatic pathways that were influenced differentially by experimental treatments. Rice-midgut vs. Corn-midgut, Rice-midgut vs. C-R-midgut and Corn-midgut vs. C-R-midgut genes indicated changes in biological pathways. In our transcriptome, the most significant changes in enzymatic pathways in Rice-midgut vs. Corn-midgut were the pentose phosphate pathway (bmor00030), oxidative phosphorylation (bmor00190), glycolysis/gluconeogenesis (bmor00010), drug metabolism-cytochrome P450 (bmor00982) and glutathione metabolism (bmor00480) (Figure 10A). Similarly for beta-alanine metabolism (bmor00410), carbon metabolism (bmor01200), fatty acid degradation (bmor00071). Glutathione metabolism (bmor00480) and metabolism of xenobiotics by cytochrome P450 (bmor00980) pathways were significantly up regulated in Rice-midgut vs. C-R-midgut (Figure 10B). In contrast a different trend was observed in Corn-midgut vs. C-R-midgut treatment in which carbon metabolism (bmor01200), pyruvate metabolism (bmor00620), glycine, serine and threonine metabolism (bmor00260), biosynthesis of amino acids (bmor01230) and purine metabolism (bmor00230) were the most significantly up regulated pathways (Figure 10C). The most common enriched pathways in all treatments were carbon metabolism, metabolism of xenobiotics by cytochrome P450, drug metabolism-cytochrome P450 and glutathione metabolism.

### 3.11. KEGG Pathway Enrichment Analysis of Male and Female Antennae

**Figure 11:**
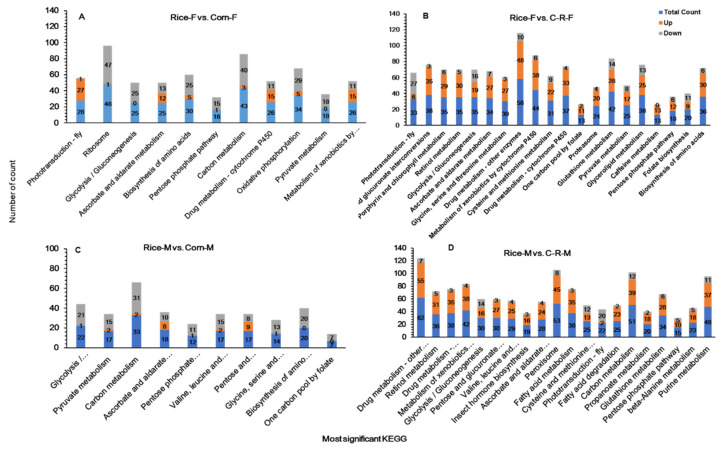
Enriched KEGG pathways for up and down regulated DEGs and the ratio of the DEG number to the total gene number in a certain pathway for Female (F) and Male (M) antennae in the Rice-F vs. Corn-F (**A**), Rice-F vs. C-R-F (**B**), (After 20 generation of selection the corn population was fed on rice for one generation (C-R-female) Rice-M vs. Corn-M (**C**) and Rice-M vs. C-R-M antennae (**D**) After 20 generation of selection the corn population was fed on rice for one generation (C-R-male) of *Spodoptera frugiperda* when reared on corn and rice plants for 20 generations.

Changes in enzymatic pathways in antennal transcriptome in Rice-Female vs. Corn-Female were ribosome (bmor03010), pentose phosphate pathway (bmor00030) and carbon metabolism (bmor01200) (Figure 11A). Similarly, pentose and glucuronate interconversions (bmor00040), retinol metabolism (bmor00830), glycine, serine and threonine metabolism (bmor00260) and cysteine and methionine metabolism (bmor00270) were the most enriched enzymatic pathways in Rice-Female vs. C-R-Female antennae, when *S. frugiperda* larvae were reared on preferred and alternate host plant for 20 generations (Figure 11B). Glycolysis / gluconeogenesis (bmor00010) was the most significant regulated pathways in Rice-Male vs. Corn-Male comparisons (Figure 11C). The transcriptome of the Rice-Male vs. C-R-Male was enriched enzymatic pathways in contrast to all other of male and female antennae of *S. frugiperda* (Figure 11D).

### 3.12. Validation of Transcriptome Data Using qPCR

The RNA-seq results were validated using RTq-PCR. The relative mRNA expression levels of 31 highly up regulated genes were selected, including 6 DEGs in Rice-midgut vs. Corn-midgut, 5 DEGs in Rice-midgut vs. C-R-midgut and four DEGs in Corn-midgut vs. C-R-midgut. Similarly, four DEGs in Rice-F vs. Corn-F, six DEGs in Rice-F vs. C-R-F, one DEGs in Rice-M vs. Corn-M and four DEGs in, Rice-M vs. C-R-M were analyzed. All the genes showed a consistent expression pattern between RTq-PCR and RNA-Seq (Figures S2 and S3).

## 4. Discussion

In herbivores insect-plant interaction, host plants have a huge impact on the dynamics and evolution of their insect parasites. Theoretically, herbivorous insects should choose host plant species that ensure their offspring’s fitness, while avoiding hosts that result in lower survival and fitness. Most herbivorous insects have evolved morphological, behavioral, physiological and genetic adaptations that enable them to specialize on one or a few plant species on which they rely for food or other resources [17,55,56,57]. Induced feeding preferences, habituation, sensitization and food aversion learning have been documented to affect host plant choice in many juvenile insects [58,59,60]. In our study, we established a corn strain of FAW on two host plants, the preferred (Corn) and alternate (Rice) for more than 20 generations and found phenotypic and genetic changes in some traits associated to adaptation to host plants. Our phenotypic results indicated that the neonate *S. frugiperda* larvae in both Y-Tube olfactometer and whole plant choice assays expressed strong feeding preference for the hosts on which they had been reared. We used newly emerged neonates that had not fed so direct host plant experience can be ruled out. Such experience can strongly affect orientation as was shown by *Spodoptera littoralis* [61]. The change in behavior in our study occurred after 5–6 generations and remained relatively stable suggesting a selection effect. However, we cannot totally rule out maternal effects as egg masses are covered by scales which may provide some host cues.

Previous studies have shown that fitness of phytophagous insects on novel hosts can increase quickly over a small number of generations [62,63], although this is not always the case [64,65]. We found *S. frugiperda* larval developmental time and pulpal weight were significantly different when reared on both plant species for more than 20 generations. In contrast to our study, previous studies have reported that the corn strain of FAW showed extended larval duration when reared on the alternative host plant, with the shortest developmental times on the preferred host plant (corn leaves) [66,67]. In our study, pupal weight increased over time when reared on rice but did vary amongst generations. Similarly variable results were reported when *S. frugiperda* were reared on corn and rice plants [68]. Host plant suitability is considered the key factor which influences larval and pupal development time in a phytophagous insects [69,70,71] which could account for some of the variation we found over time. However, the improvement overtime in our study suggests selection or plasticity effects.

The choices of gravid females for oviposition sites will severely affect their offspring performance impacting a population’s survival, thus oviposition expectancy may serve as an index of overall sensitivity of ovipositing females to adverse conditions [72,73]. We found a shift in female oviposition to rice plants as compared to corn plants when reared on rice plants. That change was evident after 4 generations and remained relatively stable for the next 16 generations. Our results are consistent with other finding in which differences in an oviposition choice were found in FAW host strains when reared on corn and rice plants for a short time [66]. Experience during the juvenile stage can affect adult behavior and host selection [74]. A number of studies with *Helicoverpa armigera* and *Spodoptera littoralis* suggest larval experience of host plants can affect host plant choice in adults, with the plant experienced during the immature stage being favored in female oviposition [75,76,77,78] but this is not always the case [79,80,81,82]. Since in our experiment the change in preference towards the initially non-preferred plant took at least 4 generation it suggests selection, but we cannot rule out gene expression.

In fact, the selection and adaptation of the host plant is decided by the insect’s internal factors and external environmental stimuli. When insects select a plant as a host, they will be spawning and feeding, and then growth and reproduction on the plant. During this process, as external environmental factors, plant volatile odors are the most important and key cues for host plant selection. At the same time, as internal factors, insect OBPs not only can selectively bind certain types of odor molecules, but also can remove toxic substances and protect the odor molecules from enzymatic degradation [83,84]. In addition to host selection, the other important aspect is host adaptation. During feeding, insect will inevitably swallow some poisonous secondary metabolites from plants. Therefore, insects have to develop an adaptation mechanism involving a series of detoxification enzymes [38,85]. Herbivorous insects also express different digestive enzymes because of the differences in nutritional value between host plants they eat [67,86,87]. We found that adaptation to host plants not only changes the survival and development but may reflect the change of transcript levels of many genes related to digestive enzymes. We found changes in gene expression related to digestive enzymes both significantly up and down in three treatment conditions (Table 2) as has been found in other insect-plant interaction [88,89,90]. Apart from nutritional necessities, herbivores insects need to deal with toxic substances from their host plants and their capability to detoxify these compounds may decide their host range. In our study, we identified many DEGs related to detoxification (Table 2). All DEGs related to detoxification enzymes such as P450s, COesterase, GSTs and UGTs were up-regulated in *S. frugiperda* larvae feeding on rice for 20 generation compared with those feeding on rice for one generation, suggesting the important roles of these genes in detoxification of chemicals during host plant adaptation, as has been found in other polyphagous insects [67,86,91]. At the same time, enzymes for detoxification are also an important factor for insect to adopt diverse host plants [17]. Therefore, identifying enzymes related to detoxification in an insect will benefit to research on insect host range and on insect pest control. Taken together, detoxification genes may all contribute to defend the caterpillar against toxic plant compounds when feed on different host plants.

GO enrichment analysis showed that DEGs related to oxidoreductase activity, hydrolase activity and cofactor binding were up- and down-regulated in all three comparisons between pairs of feeding conditions, suggesting the role of genes coding these molecular functions in host plant adaptation. Our results are similar to previous reports in which up- and down-regulation of DEGs related to oxidoreductase activity, hydrolase activity and cofactor binding were documented in insect-host response [66,92]. Heidel-Fischer and Vogel, 2015 [93] found that the change in oxidoreductase activity was largely unigenes of diverse kind of cytochrome P450, mainly implicated in xenobiotics metabolism. Similarly, it has been shown that the unigenes involved in hydrolase activity contain midgut digestive enzymes such as serine proteases and trypsins, which are mainly involved in the digestion of protein and sometimes used as an antiherbivore defense mechanism in herbivores insects [94,95,96]. These transcripts should be further studied to increase our understanding of the host range difference in FAW larvae.

Insects recognize their surrounding through different sense organs among which olfaction in antennae is crucial for the regulation of insect behaviors involved in host orientation and searching for oviposition sites [40,91]. In the present study, we identified highly expressed odorant receptors and genes in the PBP/GOBP family in Rice-Female vs. C-R-Female antennae followed by Rice-Male vs. C-R-Male antennae as compared to other groups (Table 3), suggesting that these gene families play important roles in adaptation to host plants, but additional research is needed to explore their function. In previous studies, it has been reported that the expansions of chemosensory gene families play a key role in host plant adaptation in many insect species [15,97]. Our RTq-PCR results of all selected genes showed a significant correlation between RNA-seq and RTq-PCR results, indicating the RNA-seq data (Figures S2 and S3) are reliable, as reported in previous studies [40,98,99,100]. All of the above behavioral and physiological patterns suggest that FAW can adapt to rice, in a few generations.

## 5. Conclusions

This study reports a comprehensive midgut and antennal transcriptome analysis for *S. frugiperda* after rearing on preferred and alternative host plants. Our results suggest that the fitness and development of a *S. frugiperda* on new hosts can increase quickly over a small number of generations. Furthermore, several plastic-response genes related to digestion (such as serine proteases and trypsins) and detoxification (P450s, COesterase, GSTs and UGTs) enzymes reflect the ability of this pest to adapt to a large variety of host plants. Potential adaptations for feeding on rice crops, might have contributed to the current rapid spread of this pest on rice crops in China. Similarly, the expansions of major chemosensory genes family (odorant receptor and PBP/GOBP) in antennae plays key roles for regulating insect behaviors such as host orientation, searching for oviposition sites and host plants adaptation mechanism. These results not only provide valuable insight into the molecular mechanisms to host plants adaptation of *S. frugiperda,* but may provide new gene targets for the management of this pest.

## Figures and Tables

**Table 1:** Survival and development of *Spodoptera frugiperda* after rearing on primary and alternative host plants for 20 generations. Corn- pop, Rice-pop and C-R-pop (After 20 generation the corn population was fed on rice for one generation (C-R-female).

Feeding Condition	No of 1st Instar Larvae	No of 12-Day Larvae	No Pupae	No of Emerged Adults	Larval Duration (Days)	Overall Survival (%)
Corn-Pop on rice (G-1)	400	167 (41.75%)	76 (24.25%)	47 (58.08%)	21.87 (18.25–24.26)	20.25
Corn-pop on corn-(G-20)	327	287 (87.76%)	202 (70.80%)	172 (85.15%)	18.97 (16.26–20.53)	52.59
Corn-pop on rice-(G-20)	350	321 (91.71%)	297(92.52%)	283 (95.29%)	17.03 (14.24–19.78)	80.86

**Table 2:** Summary of candidate differentially expressed genes related to digestion, detoxification and ribosome in *Spodoptera frugiperda* midgut transcriptome differentially expressed genes (log2(FC) ≥ 1, FDR < 0.05) from pairwise comparisons among the three populations of *Spodoptera frugiperda*. The Corn-midgut vs. C-R-Midgut, Rice-midgut vs. C-R-Midgut and Rice-midgut vs. Corn-Midgut.

Classification	Candidate Genes	Number of DEGs								
		Rice-Mid vs. Corn-Mid			Rice-Mid vs. C-R-Mid			Corn-Mid vs. C-R-Mid		
		Total	Up	Down	Total	Up	Down	Total	Up	Down
Digestion	*Trypsin*	56	24	32	102	45	57	31	23	8
	*carboxypeptidase*	10	7	3	21	20	1	11	9	2
	*Lipase*	15	10	5	34	29	5	5	3	2
	*Alpha amylase*	7	3	4	7	6	1	2	2	0
	*cysteine protease*	3	0	3	15	5	8	1	1	0
	*serine protease*	4	2	2	8	2	6	0	0	0
	*Trypsin Inhibitor*	8	6	2	12	10	2	2	2	0
Detoxification	*P450s*	25	17	7	56	34	22	15	7	8
	*CEs*	26	18	8	43	32	11	6	4	2
	*GSTs*	14	11	3	14	4	10	1		2
	*UGTs*	18	5	13	30	6	24	4	3	1
	*ABC transporters*	7	4	3	26	18	8	0	0	0
Ribosomal	*Ribosomal protein*	3	0	3	23	2	21	1	0	1

**Table 3:** Summary of candidate differentially expressed genes related to chemosensation in male and female antennal transcriptome of Spodoptera frugiperda, Rice-female vs. Corn-female, Corn-female vs. C-R-female (After 20 generation the corn population was fed on rice for one generation (C-R-female)) and Rice-male vs. Corn-male, Corn-male vs. C-R-male antennae after feeding on Corn and rice plants for 20 generations or on rice for one generation (C-R).

Candidate Genes	Number of DEGs											
	Rice-F vs. Corn F			Rice-F vs. C-R-F			Rice-M vs. Corn-M			Rice-M vs. C-R-M		
	Total	Up	Down	Total	Up	Down	Total	Up	Down	Total	Up	Down
*Insect pheromone-binding family*	9	1	8	7	4	3	10	0	10	5	3	2
*PBP/GOBP family*	4	3	1	25	21	4	6	0	6	4	0	4
*Olfactory receptor*	1	0	1	0	0	0	0	0	0	0	0	0
*Odorant receptor*	6	4	2	22	22	0	4	4	0		14	3
*Chemosensory receptor*	1	1	0	2	2	0	0	0	0	0	0	0

## Supplementary Materials

The following are available online at https://www.mdpi.com/article/10.3390/ijms221910284/s1.
