# Supplementary material for: Behavioral and Physiological Plasticity Provides Insights into Molecular Based Adaptation Mechanism to Strain Shift in Spodoptera frugiperda
Source: Int J Mol Sci. 2021 Sep 24;22(19):10284. doi: 10.3390/ijms221910284 (PMC8508907; doi:10.3390/ijms221910284)
Supplement: Supplementary file 1 [file ijms-22-10284-s001.zip › ijms-1366096-supplementary.pdf]

Table S1. Summary of the sequencing data of Midguts

Samples	Raw reads	Clean reads	Clean bases	Error rate	Q20	Q30	GC %
**Rice_Female1**	46010308	45059750	6.76G	0.02	98.04	94.43	45.35
**Rice_ Female2**	46086786	44824336	6.72G	0.02	98.06	94.3	44.58
**Rice_ Female3**	47322450	46341508	6.95G	0.02	98.07	94.45	45.33
**Corn_ Female1**	48012490	47136864	7.07G	0.03	97.95	94.19	45.82
**Corn_ Female2**	45554128	44784514	6.72G	0.02	98.04	94.41	46.49
**Corn_ Female3**	50361454	49523244	7.43G	0.03	97.95	94.18	46.76
**C_R_ Female1**	47224770	46241700	6.94G	0.02	97.97	94.26	44.92
**C_R_ Female2**	46929568	46003002	6.9G	0.02	98.03	94.42	45.7
**C_R_ Female3**	50059668	49058420	7.36G	0.02	97.99	94.33	45.6
**Rice_Male1**	45689210	44508098	6.68G	0.02	98.01	94.38	45.15
**Rice_ Male2**	47787892	46710430	7.01G	0.02	98.03	94.32	44.65
**Rice_ Male3**	47005582	46047096	6.91G	0.02	97.97	94.24	44.63
**Corn_ Male1**	45983330	45252070	6.79G	0.03	97.96	94.22	45.44
**Corn_ Male2**	46490752	45791600	6.87G	0.02	97.96	94.26	46.29
**Corn_ Male3**	45921054	45269004	6.79G	0.02	98.06	94.51	47.12
**C_R_ Male1**	47338076	46299766	6.94G	0.02	97.96	94.29	46.76
**C_R_ Male2**	46327828	45381322	6.81G	0.02	98.1	94.53	45.11
**C_R_ Male3**	47456696	46228984	6.93G	0.02	98.05	94.42	45.2

Table S2. Summary of the sequencing data of Midguts

Samples	Raw reads	Clean reads	Clean bases	Error rate	Q20	Q30	GC %
**Rice_Midgut1**	41418168	40553312	6.08G	0.02	98.1	94.55	48.3
**Rice_ Midgut2**	46126022	45213780	6.78G	0.02	98.05	94.48	48.56
**Rice_ Midgut3**	42695610	41842840	6.28G	0.02	97.99	94.25	48.05
**Corn_ Midgut1**	50552144	49495892	7.42G	0.02	98	94.39	48.92
**Corn_ Midgut2**	46716724	45436592	6.82G	0.02	98.14	94.58	45.71
**Corn_ Midgut3**	45896862	44783588	6.72G	0.02	98.25	94.88	45.96
**C_R_ Midgut1**	45337686	44215144	6.63G	0.02	98.06	94.43	46.6
**C_R_ Midgut2**	48099646	46903402	7.04G	0.02	98.07	94.52	47.16
**C_R_ Midgut3**	46410788	45270418	6.79G	0.02	98.1	94.54	45.73

Table S3. List of primers used in this study for qRT-PCR

Primers used for qRT-PCR	ID number	Sense primers	Anti-sense primers	Size of the amplicon
OR15	GSSPFG00004022001.5	AGTCAAGTTGTCGCGCCTAT	GGGACTCCCGATCTTCATCAC	103
OR35	GSSPFG00031639001.3	TGGTGAGGTACATGCGTGA	GGGGCGCAATATGGGTATGA	102
OR1	GSSPFG00012479001.6	GAGTACGACGATGCGACGAT	GGGAGACGAAGCTCTCGAAC	97
OR17	GSSPFG00025871001.3	TGGCAGCCACCTCTACCTAT	GTGGCACAGCAACACAAACA	104
novel.5605	novel.5605	CCATCAGCCGTTCACCAAGA	GCAAACTGCGTCATCTCGTC	126
OR8	GSSPFG00031395001.6	TGATGGCTCCTTGTTGGCTT	GAGAGCGGTTTGTTAGCCCT	148
OR11	GSSPFG00004819001.3	ACAATAACTACGCTGCCGGT	CCAGCCACTAAGAGTCGCAA	140
OR15	GSSPFG00004022001.5	TCGTGATGTGCGGTCTTCTT	GACTAGCCTTTCGCTTCCGT	140
OR45	GSSPFG00035294001.4	TCTTCAAAGACAACGAGGAGCA	TTCCGTCACTCGTGTTCTGG	90
OR5	GSSPFG00015711001.5	ATGCGCCACTGATAGACAGG	CATCGACATCAGGCACCGTA	73
OR4	GSSPFG00035258001.3	TCAACTTCGCTCCGCTGATT	CACGCAAATTCTCCCAACCG	132
OR51	GSSPFG00010075001.3	GCGATCTACGGTGGAGGTTG	CAACGCTACACCACGCTCTA	150
OR56	GSSPFG00032130001.3	ACTGTGTACCTCAGGGCGTA	ACGTGAGCATGGTGATCCTG	112
OR44	GSSPFG00003382001.6	CTGTCGATGACACAACCGGA	TCTTCCGTGAGCTGGTTTCC	116
OR11	GSSPFG00004819001.3	TGGAAGAGGTCGCTGTCAAG	AATGAAGAGCGCCATTCCGA	104
Cytochrome P450	GSSPFG00019627001	TATCGGTTGGCATTGGTCGT	ATACTGCGGCATTCGAGAGG	116
Cytochrome P450	GSSPFG00033942001	TTGAAGCGGATGCCGTACTT	TATGGGCGCTACGAAGGAAC	147
Trypsin	GSSPFG00015539001	TTACTGCTGCACACTGCTCA	GTCTCAAAAGCCCAAGTGCG	72
novel.3161	novel.3161	CCAGCAGCCTCTGAGATACG	AACAGCGTGATCACTAGCCC	107

Carboxylesterase family	GSSPFG00035313001.4	ATGCCAGTCCTATTGCGGAG	AATGGGCCCTCGGAAATGTT	71
Cytochrome P450	GSSPFG00019609001	CGGCGGAGACTGTGATGTAA	CTGAACAGGGAGTGCTCGTT	114
UGT40Mc	GSSPFG00021628001.3	ATCGTTTGACGAAGCCAGGT	AAAGGTACGTGGAGTGCTGG	103
Carboxylesterase family	GSSPFG00021093001.4	ACGCACCGATGAGTGTCATT	TCTCATTGCGCCTTCCATGT	86
Trypsin	GSSPFG00024088001	AAATGTTGTGCGCTGGTTGG	CGAAAGAGTAGACGCCGACA	105
novel.3161	novel.3161	CCAGCAGCCTCTGAGATACG	AACAGCGTGATCACTAGCCC	107
Trypsin	GSSPFG00031776001	TGTTCTGGTGTAGTGGTCGC	ACACGGTGGTGTCGTCTTTT	118
UGT39-01	GSSPFG00035503001.3	AGTTAGCCCGTAGGGGTCAT	CTCGTCCAAGAACGTCCCAA	120
Trypsin	GSSPFG00031776001	TGTTCTGGTGTAGTGGTCGC	ACACGGTGGTGTCGTCTTTT	118
Trypsin	GSSPFG00009802001	TTATGTGCGGGAAGGCAAGG	CCACATTGAGGTCCTCCGAA	110
Trypsin	GSSPFG00034812001	AGCACTACAGCATCAAGCGT	CGTACGTGTAGGTCCTCGTG	107
S30	AF400225.1	CACCCTCGGTGTTAGACGTT	CCACCGGGAAAGTGATACTGT	119
GAPDH	KC262638.1	CGGTGTCTTCACAACCACAG	TTGACACCAACGACGAACAT	111

Figure S1. Highly expressed DEGs in the antennae (A) and midguts (B) of Rice and Corn population

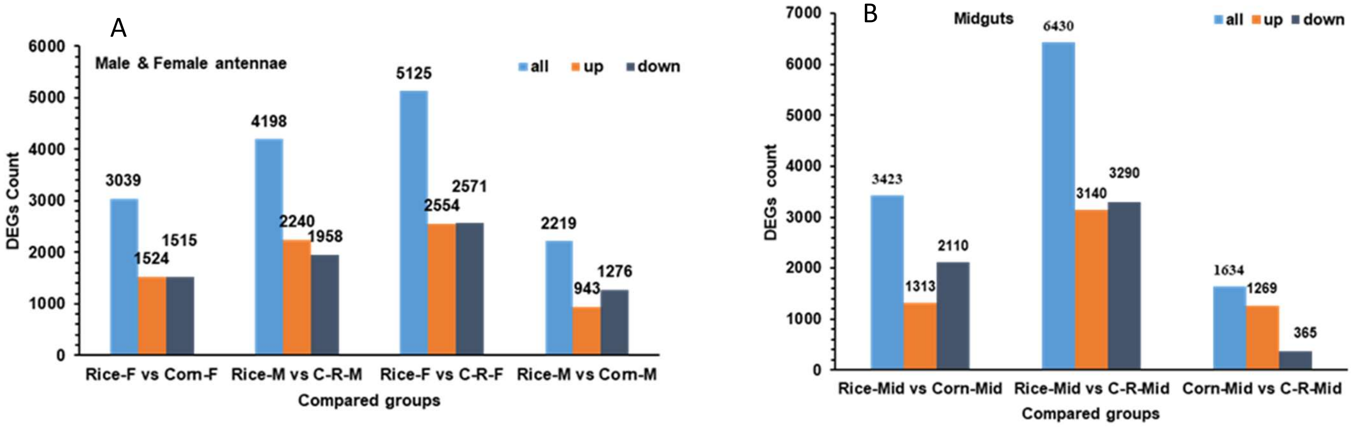

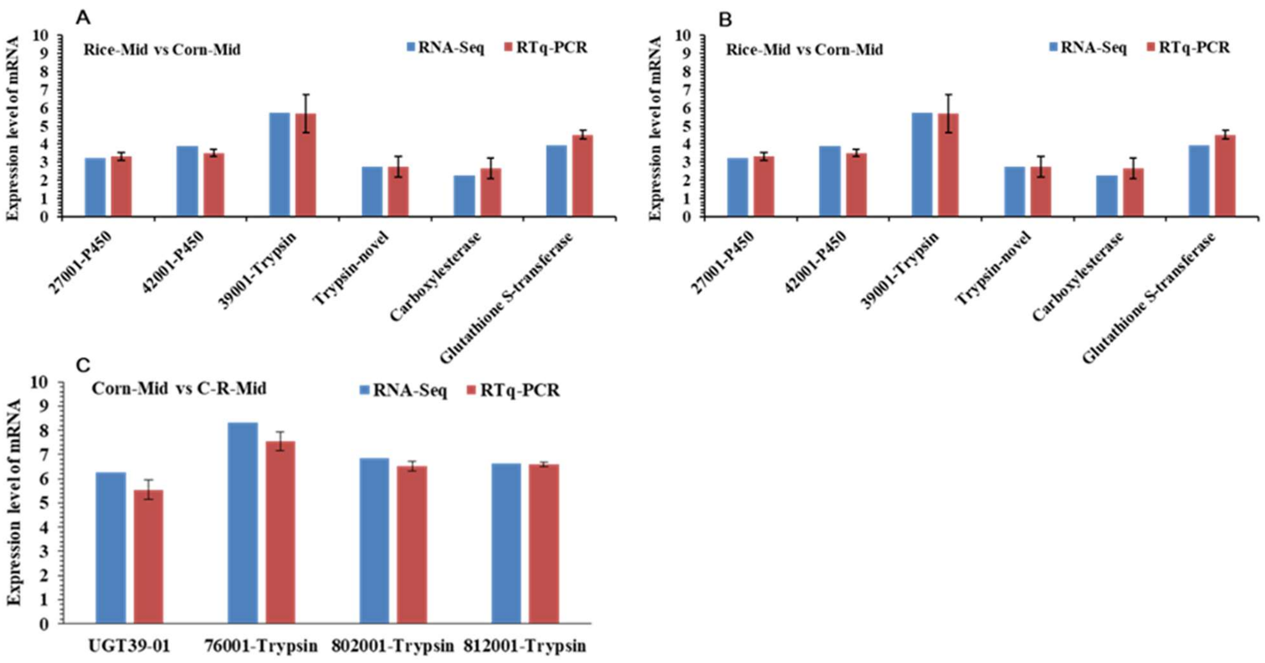

Fig. S2. Gene expression levels of differentially expressed genes through RNA-seq and qRT-PCR analysis in the Rice-Mid vs Corn-Mid (A), Rice-Mid vs C-R-Mid (B), Corn-Mid vs C-R-Mid (C) (After 20 generation of selection the corn population was fed on rice for one generation (C-R-midgut) of *Spodoptera frugiperda* when reared on corn and rice plants for 20 generations.

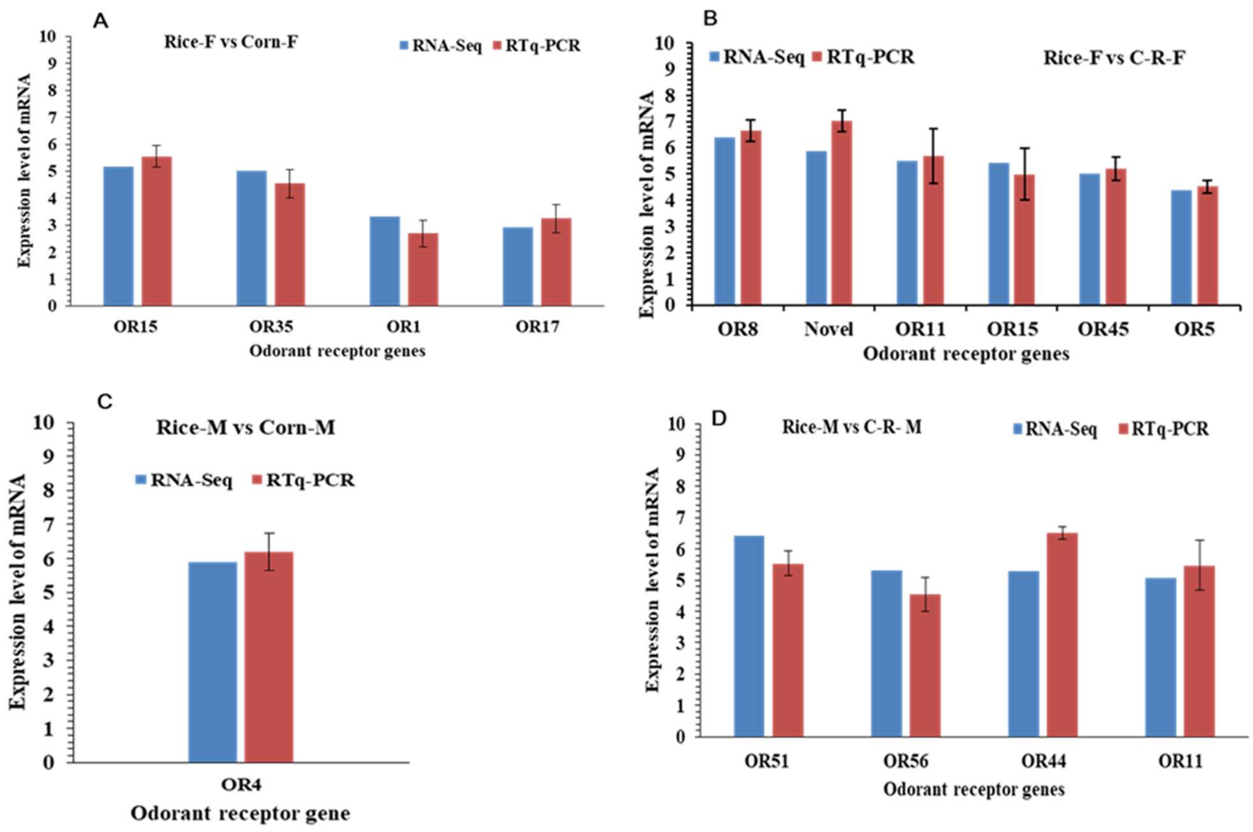

Fig. S3. Gene expression levels of differentially expressed genes through RNA-seq and qRT-PCR analysis in Female (F) and Male (M) antennae of Rice-F vs Corn-F (A), Rice-F vs C-R-F (B), (After 20 generation of selection the corn population was fed on rice for one generation (C-R-female)Rice-M vs Corn-M (C) and Rice-M vs C-R-M (After 20 generation of selection the corn population was fed on rice for one generation (C-R-male)of *Spodoptera frugiperda* when reared on corn and rice plants for 20 generations.
